# Inflammatory Biomarkers in Cancer

**DOI:** 10.1155/2016/7282797

**Published:** 2016-10-23

**Authors:** Tomoki Nakamura, Czar L. Gaston, Krishna Reddy, Shintaro Iwata, Jun Nishio

**Affiliations:** ^1^Department of Orthopaedic Surgery, Mie University Graduate School of Medicine, Mie, Japan; ^2^Oncology Service, The Royal Orthopaedic Hospital, Birmingham, UK; ^3^Department of Orthopaedic Surgery, The University of Cincinnati, Cincinnati, OH, USA; ^4^Department of Orthopaedic Surgery, Chiba Cancer Center, Chiba, Japan; ^5^Department of Orthopaedic Surgery, Fukuoka University Graduate School of Medicine, Fukuoka, Japan

In this special issue, we investigate the relationship between inflammation and cancer. Inflammation plays a critical role in the progression of cancers. Several biomarkers have been reported as useful marker for clinical cancer behavior. In this issue, authors reported possible biomarker for the development and prognosis of several cancers including esophageal cancer, breast cancer, colorectal cancer, ovarian cancer, and myelofibrosis.

P.-C. Chen and J.-F. Feng looked at 323 patients with resectable esophageal squamous cell carcinoma (ESCC) and proposed a prognostic staging system for cancer specific survival based on markers of inflammation. Patients with high levels of C-reactive protein, neutrophil to lymphocyte ratio, and platelet count to lymphocyte ratio (stage I3) had significantly poorer survival compared to patients with one or more of these inflammatory parameters within normal limits. The predictive value of their system was maintained in different TNM stages of ESCC and would be a useful tool for clinicians.

M. Zajkowska et al. compared levels of VEGF, M-CSF, and CA 15-3 of 120 patients with breast carcinoma with a control group composed of 60 patients with benign breast tumors and 60 healthy volunteers. They found that expression of VEGF had the highest sensitivity and specificity in differentiating stage 1 breast cancer from the control group, while a combination of VEGF and CA 15-3 had the highest sensitivity and specificity in detecting stage III and stage IV breast cancer. Their results contribute to the growing evidence for the utility of these biomarkers as diagnostic tools in breast cancer.

M. Rutka et al. evaluate the diagnostic accuracy of five different fecal markers for detection of precancerous and cancerous lesions of the colorectum in a prospective, colonoscopy study and report sensitivity and specificity of each of these markers. They established that sensitivity of M_2_ Pyruvate Kinase, iFBOT, and Hb/Hp complex to be high in colorectal carcinoma but decreased in adenomas ≥ 1 cm in size. They recommend combined use of M_2_PK, iFBOT, and FC for detecting larger adenomas. The authors suggest these noninvasive fecal screening tests for low risk patients or as a part of a two-step screening process with colonoscopy being the gold standard.

Z. Liu et al. addressed whether the expression of Notch3, a type of Notch receptor which activates the PI3K/Akt/mTOR signaling pathway, and ribosomal S6 kinase (S6K), a downstream effector of the PI3K/Akt/mTOR pathway, correlated with the clinical features and prognosis in ovarian epithelial cancer. Their results showed that Notch3 and pS6K expression associated with clinical stage and pathological grading, resulting the association with poorer survival. They concluded that Notch3 and pS6K are potential biomarkers and therapeutic targets in ovarian epithelial cancer.

D. Sollazzo et al. investigated whether the concentration of circulating calreticulin in plasma calreticulin in healthy subjects and in patients with myelofibrosis (MF) differs. The authors demonstrated that the concentration of calreticulin is higher in patients with MF compared to healthy subjects. In contrast, circulating calreticulin levels and mutation status or JAK2V617F burdens were not correlated. The authors conclude that high circulating calreticulin levels in MF reflects chronic systemic inflammation.

We hope that researchers enjoy the reading of this special issue related to inflammation and biomarkers in cancer. Undoubtedly, the presence of an association between systemic inflammation and a poor prognosis has been established.

*Tomoki Nakamura* *Tomoki Nakamura* *Czar L. Gaston* *Czar L. Gaston* *Krishna Reddy* *Krishna Reddy* *Shintaro Iwata* *Shintaro Iwata* *Jun Nishio* *Jun Nishio*

